# A STUDY COMPARING CHEMICAL PEELING USING MODIFIED JESSNER'S SOLUTION AND 15%TRICHLOROACETIC ACID VERSUS 15% TRICHLOROACETIC ACID IN THE TREATMENT OF MELASMA

**DOI:** 10.4103/0019-5154.48985

**Published:** 2009

**Authors:** Omar Soliman Safoury, Nagla Mohamed Zaki, Eman Ahmad El Nabarawy, Eman Abas Farag

**Affiliations:** *From the Department of Dermatology, Cairo University, Cairo, Egypt*

**Keywords:** *Classic Jessner's solution*, *chemical peeling*, *melasma*, *modified Jessner's solution*, *trichloroacetic acid*

## Abstract

**Background:**

Melasma is a symmetric progressive hyperpigmentation of the facial skin that occurs in all races but has a predilection for darker skin phenotypes. Depigmenting agents, laser and chemical peeling as classic Jessner's solution, modified Jessner's solution and trichloroacetic acid have been used alone and in combination in the treatment of melasma.

**Objectives:**

The aim of the study was to compare the therapeutic effect of combined 15% Trichloroacetic acid (TCA) and modified Jessner's solution with 15% TCA on melasma.

**Materials and Methods:**

Twenty married females with melasma (epidermal type), with a mean age of 38.25 years, were included in this study. All were of skin type III or IV. Fifteen percent TCA was applied to the whole face, with the exception of the left malar area to which combined TCA 15% and modified Jessner's solution was applied.

**Results:**

Our results revealed statistically highly significant difference between MASI Score (Melasma Area and Severity Index) between the right malar area and the left malar area.

**Conclusion:**

Modified Jessner's solution proved to be useful as an adjuvant treatment with TCA in the treatment of melasma, improving the results and minimizing postinflammatory hyperpigmentation.

## Introduction

Melasma is a symmetric progressive hyperpigmentation of the facial skin that occurs in all races but has a predilection for darker skin phenotypes. Melasma has been associated with hormonal imbalance, sun damage, and genetic predisposition. Clinically, melasma can be divided into centrofacial, malar, and mandibular, according to the pigment distribution on the skin. By Wood's light examination, melasma can be classified into epidermal, dermal or mixed type.[1]

Many depigmenting agents and other therapies such as chemical peeling are used for treating melasma, in the form of monotherapy or combined therapy.[2,3] The most commonly used peeling agents are alpha-hydroxy-acids, resorcinol, Jessner's solution, and trichloroacetic acid.[4] TCA is the most popular peeling agent used in different concentrations. It has the broadest spectrum of indications.[5]

The Jessner's-trichloroacetic acid peel is a procedure developed by Dr. Gray Monheit (USA) to produce a safe, effective medium-depth chemical peel for the treatment of photoaged skin, actinic keratoses, and superficial acne scars.[6]

The aim of this work was to compare the efficacy of 15% TCA peeling as against the combined modified Jessner's and 15% TCA peeling in the treatment of melasma.

## Materials and Methods

Twenty married females with melasma, with a mean age of 38.25 years, were included in this study. All were of skin type III or IV. The duration of the melasma ranged from six months to 15 years, with a mean of 7.6 years.

All the participants were subjected to Wood's light to determine the type of melasma (epidermal from dermal or mixed). Only patients with epidermal type were included in this study. Melasma Area and Severity Index (MASI) score of the right and left cheeks were calculated for each patient at baseline, at the beginning of each peeling session, and at the end of follow up, along with photography. The patient did not know the types of peeling agents (written in the consent). Although the peeling was done by one doctor and the MASI score was done by another doctor, it was impossible to blind the two peeling agents because of the very characteristic odor and the absence of frost in the modified Jessner's solution. The MASI score was calculated by the following formula: Adding the sum of the severity ratings for darkness and homogeneity, multiplied by the numerical value of the areas involved and by the percentage of the malar area (correspond to 30% of the total face).[7]

A numerical value assigned for the corresponding percentage area (A) involved is as follows: (0) no involvement; (1) <10% involvement; (2) 10-29% involvement; (3) 30-49% involvement; (4) 50-69% involvement; (5) 70-89% involvement; and (6) 90-100% involvement.

The darkness of the melasma (D) is compared to the normal skin and graded on a scale of 0 to 4 as follows: (0) for normal skin color without evidence of hyperpigmentation; (1) for barely visible hyperpigmentation; (2) for mild hyperpigmentation; (3) for moderate hyperpigmentation and (4) for severe hyperpigmentation. The homogeneity of the hyperpigmentation (H) was also graded on a scale of 0 to 4 as follows:(0) for normal skin color without evidence of hyperpigmentation; (1) for specks of involvement; (2) for small patchy areas of involvement <1.5 cm diameter; (3) for patches of involvement >2 cm diameter and (4) for uniform skin involvement, without any clear areas.

### Inclusion criteria

- Adults >18 years old
- Clinical diagnosis of melasma
- Mental capacity to give informed consent

### Exclusion criteria

- Pregnant females and females on oral contraceptive pills
- Participants with a history of hypertrophic scars or keloids
- Participants with dermal or mixed melasma
- Participants with recurrent herpes infection
- Presence of cutaneous infection.

#### Preparation:

The participants were primed two weeks before starting the peel with Adapalene 0.1% gel, once/day.[8]

#### Sun block:

The participants were strictly instructed to apply 10% zinc oxide as a sun block, during and after therapy. Ten percent zinc oxide protects against UVA, UVB and visible light.[9] Sunscreens that primarily block UVB radiation (290-320nm) are unsatisfactory, because longer wavelengths UVA and visible radiation (320-700nm) also stimulate melanocytes to produce melanin.[10]

#### Preparation of the peeling agents:

Fifteen percent TCA (Delasco) was prepared by adding 85 ml of distilled water to 15gm of TCA (weight to volume preparation). Modified Jessner's solution (Delasco) preparation was formed with 8% citric acid (weight to volume), 17% lactic acid (weight to volume), 17% salicylic acid (weight to volume), and ethanol anhydrous.

#### Application of the peeling agents:

After cleaning and degreasing with alcohol, Modified Jessner's solution was applied before 15% TCA application to the left malar area only, until the appearance of erythema. Fifteen percent TCA was applied in one uniform coat to the whole face, for the sake of the participants, until frosting. The participant was then allowed to wash her face with alkaline soap. Then the participants applied mild corticosteroid cream for two days only and sun block continuously. Peeling settings was done every 10 days (unless there was persistence of erythema or peeling, in which case, we waited until they subsided). Stopping peeling was done when clearance of melasma occurred or after a maximum of eight sessions, if the melasma did not completely disappear.

#### Follow up evaluation (after eight weeks):

All the participants were evaluated for recurrence of melasma, guided by the MASI score.

### Data management and statistical analysis

Data analysis and calculations to produce a graphic presentation of important results were done using the statistical software package SPSS 12. Data were tabulated and statistically analyzed to evaluate the difference between the groups under study, with regard to the various parameters. Together, correlations were tried in between the essential studied parameters. The statistical analysis included; the arithmetic mean, standard deviation, standard error, and Student's “t” Test. The probability (*P*) for the “t” value with degrees of freedom, obtained from the statistical Tables, was directly supplied by the computer.

#### Significance of results:

Non significant (NS) if *P* > 0.05, Significant (S) if *P* < 0.05, High significant (HS) if *P* < 0.01.

## Results

### Clinical results in the right malar area (TCA only)

**Figure 1:**
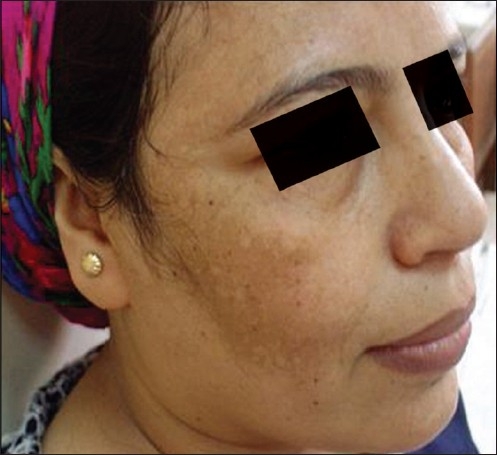
Right side before

**Figure 2:**
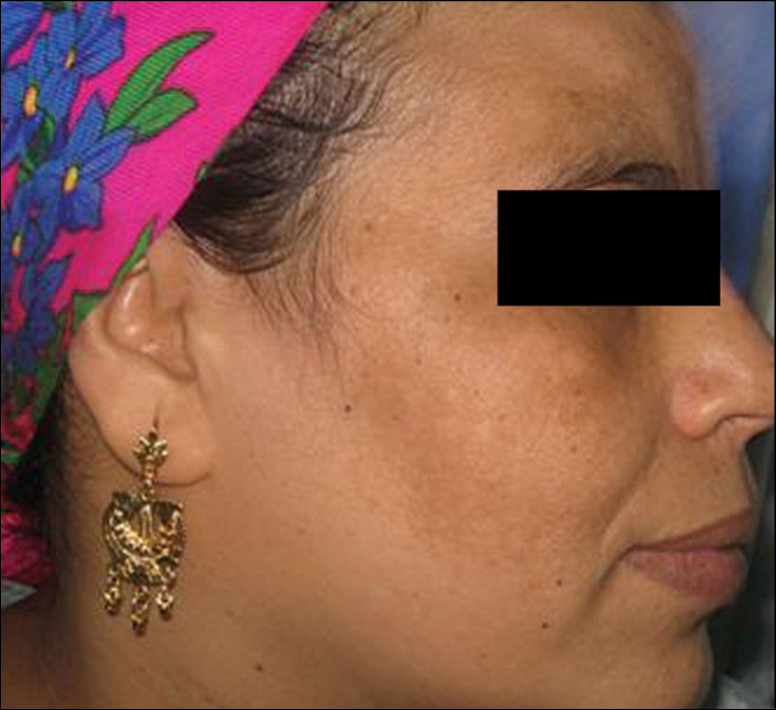
Right side after

The average (mean) MASI score in the right malar area before treatment was 4.460 ± 1.571, whereas after treatment (at the end of eight peeling sessions), the average MASI score changed to 2.040 ± 1.326. So, the average decrease in MASI score was 2.420 [Figure 1, Figure 2]. This represents a 54.26% decrease and was statistically highly significant (*P* = 0.000) (*P* value is highly significant ≤0.01).

At the end of the follow up period (eight weeks), the average MASI score raised to 2.270 ± 1.400. The final average decrease in MASI score was 2.190. This represents a 49.1% decrease and was statistically highly significant (*P* = 0.000).

### Clinical results in the left malar area (Combined TCA and modified Jessner's solution)

**Figure 3:**
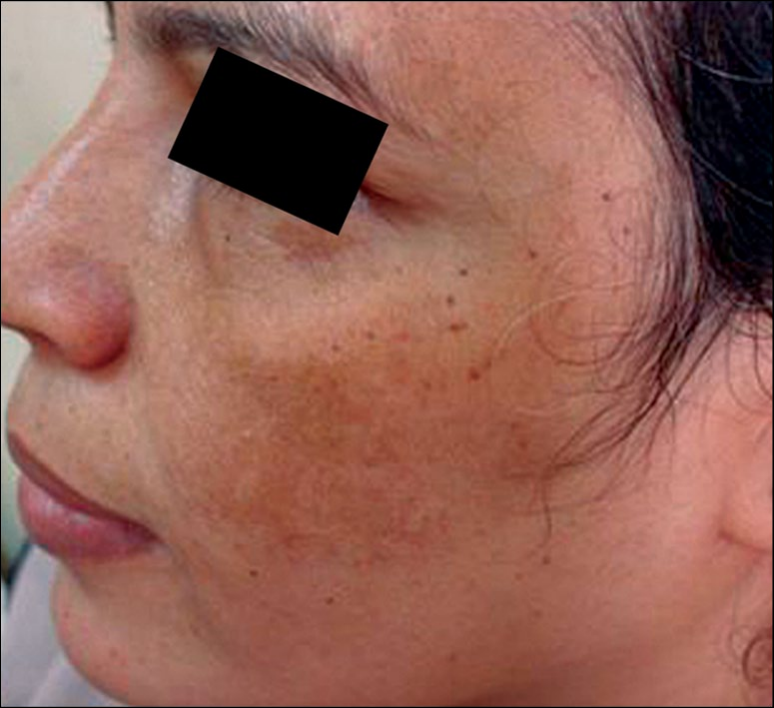
Left side before

**Figure 4:**
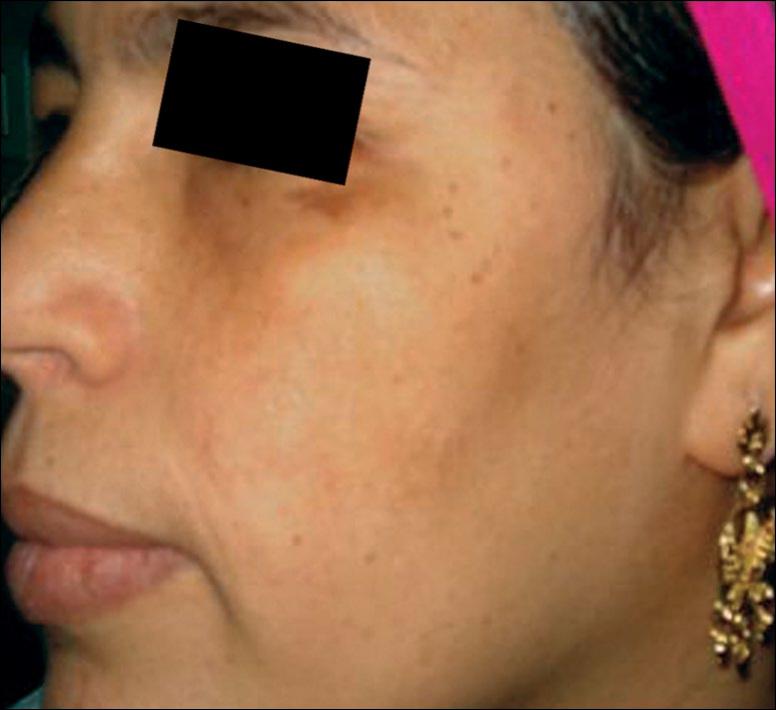
Left side after

The average (mean) MASI score in the left malar area before treatment was 4.350 ± 1.468, whereas after treatment (at the end of eight peeling sessions), the average MASI score changed to 1.230 ± 0.808 [Figure 3, Figure 4]. So, the average decrease in MASI score was 3.120. This represents a 71.72% decrease and was statistically highly significant. (*P* = 0.000) (*P* value is highly significant if ≤0.01).

At the end of the follow up period (eight weeks), the average MASI score raised to 1.670 ± 1.175; so the final average decrease in MASI score was 2.680. This represents a 61.6% decrease and was statistically highly significant (*P* = 0.000).

### Comparisons of MASI score in both groups

**Figure 5:**
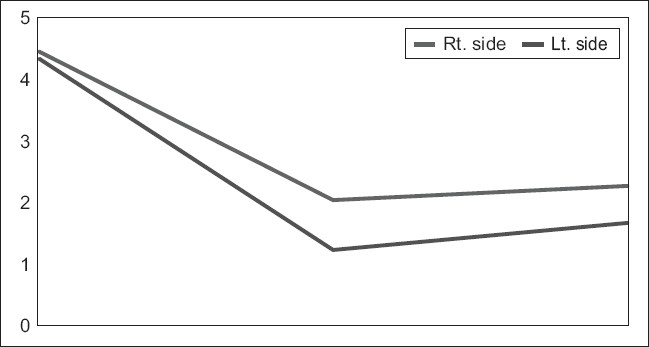
MASI score over the course of the study in both malar areas at the end of peeling and follow up

Comparison between the mean MASI score after peeling in both cheeks showed highly significant *P* value (0.000) [Figure 5]. Comparison between the MASI score at the end of follow up showed a less highly significant *P* value (0.002).

### Comparison of clinical side effects in both groups

Discomfort: Chi square = 14.40, *P* value <0.01 highly significant [Table 1].

**Table 1:** Side effects during peel in both malar areas

Side effects	Right malar	Left malar	*P* value
Swelling	2 patients (10%)	2 patients (10%)	>0.05
Erythema	5 patients (25%)	5 patients (25%)	>0.05
Discomfort	4 patients (20%)	16 patients (80%)	<0.01
Post inflammatory hyperpigmentation	2 patients (10%)	No patient (0%)	>0.05
Folliculitis/Acne	5 patients (25%)	5 patients (25%)	>0.05

Postinflammatory hyperpigmentation (PIH): Chi square = 2.11, *P* value >0.05 non-significant.

Swelling, erythema and acne: *P* value >0.05 non-significant.

## Discussion

Melasma is an acquired hyperpigmentation caused due to an increase in the amount of melanin within melanocytes. Most often, it affects the forehead, malar eminences, upper lip, and chin. The hyperpigmented patches are usually symmetrical and have a sharp irregular border. Histologically, three forms exist (epidermal, dermal, and mixed). The epidermal type is the most responsive to treatment.[11] Melasma is a cosmetic problem that sometimes causes great emotional suffering. Women with melasma have decreased health-related quality of life (HRQoL).[12]

The therapy for melasma has always been challenging and discouraging. The current treatments include hypopigmenting agents, chemical peels and laser.[13] It is important to avoid exposure to the sun or to ultraviolet lamps and to use broad-spectrum sunscreens. Several hypopigmenting agents such as topical hydroquinone (2 to 4%) alone or in combination with tretinoin (0.05 to 0.1%) have been used with differing results. Topical azelaic acid (15 to 20%) can be as efficacious as hydroquinone. Kojic acid, alone or in combination with glycolic acid or hydroquinone, has shown good results, due to its inhibitory action on tyrosinase. Laser therapies have not produced completely satisfactory results, because they can induce hyperpigmentation and recurrences can occur.[14]

Chemical peeling has a low rate of complications and is popular due to the low costs involved and to a technique which is easy to learn.[15] Chemical peels useful in treating melasma are trichloroacetic acid, Jessner's solution, [alpha]-hydroxy acid preparations, and salicylic acid, alone or in various combinations. These have been found to be particularly useful for melasma of varying severity.[12]

The gold standard for chemical peeling agents is TCA. It has been well studied and is versatile in its ability to create superficial, medium-depth and deep peels. It is stable, inexpensive and causes no systemic toxicity. It is easy to perform, as the peel depth correlates with the intensity of skin frost and there is no need to neutralize a TCA peel.[16]

In the treatment of melasma, a few studies were done using TCA as a chemical peeling agent. Some studies used TCA alone (up to 30%) in the classic full face peeling technique,[17] or focally using higher concentrations of TCA up to 50%.[18] Other studies used TCA in combination with topical vitamin C.[19] Some other studies used TCA in combination with other treatment modalities, such as Q- switched Alexandrite laser or pulsed dye laser (PDL).[20] The presence of few studies is probably due to the fact that melasma is more prevalent in darker complexioned individuals in whom there is a higher tendency for pigmentary changes to develop. Hyperpigmentation is the commonly observed side effect, with superficial peels, especially with TCA, in people living in tropical countries with intense ultraviolet exposure.[21]

When the dermatologists initially began using TCA, the usual strength was 20% to 25%. Trichloroacetic acid (TCA) turned out to be not as predictable as phenol. With phenol peels, there is uniform penetration to a certain level, then the action stops. This is not the case with TCA: there are “hot spots” where the TCA will penetrate deeper for no apparent reason. These hot spots are less troublesome as the concentration is decreased. The results with TCA are coat dependent (i.e., dependent on the number of layers applied), whereas the results with alpha-hydroxy acids are time dependent. The more coats that are used the deeper the peel; therefore, multiple coats of a 15% TCA can mimic the results of one or two coats of 35% TCA.[22] These diluted peels take more time and more coats; so we have more time to watch the blanch develop and we can discontinue applications at the desired level. The weaker strengths of trichloroacetic acid gives us more time to observe these color changes in the skin, as they occur.

Gary Monheit has popularized the combination peel using the classic Jessner's solution combined with TCA, to achieve a more uniform penetration and an excellent peel with a low, safe concentration of TCA. First, the skin is prepared with an acetone/alcohol solvent and a cleanser (Septisol), before the application of Jessner's solution. This is followed with TCA 35%.[6,22,23] The efficacy of the chemical peeling performed with Jessner's solution and 35% TCA in the treatment of melasma was evaluated in a study that included 24 patients with melasma and proved to be an effective and reliable therapeutic modality for the treatment of melasma.[25]

In our study, based on the above data, we tried to avoid hyperpigmentation, which is a commonly observed side effect with 35% TCA, by using lower concentration of TCA (15% only). We also used modified Jessner's solution (lactic acid 17%, salicylic acid 17%, and citric acid 8% in ethanol 95%) without resorcinol, instead of the classic Jessner's solution, to avoid possible allergic reactions and hyperpigmentation problems, which may be created by resorcinol, especially in skin types V and VI.[25]

Adapalene gel and TCA (15%) were applied on both sides of the face. Therefore, the difference in the MASI score can only be explained by the addition of modified Jessner's solution in the left malar area. With modified Jessner's solution as a pretreatment keratolytic, the epidermal barrier is altered prior to TCA peeling, to help more rapid and uniform uptake.[26]

During the peeling sessions, we noticed that frosting developed earlier in the left side. Also, peeling started earlier by several hours in the left side than in the right side (peeling started on the third day). Again, these differences can only be explained by the addition of modified Jessner's solution in the left malar area.

As regards the side effects, they were the same in both sides, in the form of erythema, swelling, acne and folliculitis. Discomfort due to more burning sensation was more obvious with modified Jessner's solution, which subsided as frosting was completed, while PIH developed only with 15% TCA in two (10%) patients [Table 1].

On reviewing the literature, we found that there is one study which tried to evaluate the efficacy of chemical peeling performed with Jessner's solution and 35% TCA in melasma. Twenty four participants were included in the study. After a follow-up period of six months, the pigmentation degree and the extent of the melasma lesions were found to be reduced in 83.2% and 70.8% of the participants consecutively. The overall success rate was determined to be about 100% in epidermal and mixed types of melasma, whereas it only reached 42.8% in the dermal type.[24] This is, to our knowledge, the first study that compares the effect of combined TCA (15%) and modified Jessner's solution with TCA (15%) alone in the treatment of melasma.

By using modified Jessner's solution combined with TCA (15%), we decrease the risk of PIH that occurs commonly in dark races, following the TCA peels.

From the historical point of view, in 2000, Cook total body peel™ was introduced as the first combined peel. It was developed for reshaping the contour of chin, neck and tightening of the skin. It was designed to be safe for nonfacial skin. It is made of combination of 40% TCA and 70% glycolic acid gel.[27]

## Conclusion

In our study, modified Jessner's solution proved to be useful as an adjuvant treatment with TCA in the treatment of melasma, improving the results and minimizing PIH.

## Recommendations

We recommend more studies on combined peel, using modified Jessner's solution with different concentrations of TCA (15% or others) and using larger samples of people and in other pigmentary disorders.
